# Nomogram analysis of factors associated with prognosis in patients with sepsis-associated acute kidney injury: a case-control study based on LASSO regression

**DOI:** 10.3389/fmed.2026.1838034

**Published:** 2026-06-05

**Authors:** Kabinuer Keyimu, Palida Abulizi, Julaiti Rouzhahong, Chen Wei

**Affiliations:** Department of Geriatrics, The First Affiliated Hospital of Xinjiang Medical University, Urumqi, China

**Keywords:** LASSO regression, nomogram, prediction model, prognosis, sepsis-associated acute kidney injury

## Abstract

**Background:**

Sepsis-associated acute kidney injury (SA-AKI) is one of the most common and serious complications of sepsis, characterized by high mortality, prolonged hospital stay, substantial healthcare burden, and progression to chronic kidney disease in approximately 30% of survivors. Early identification of high-risk patients and accurate prediction of their prognosis are of great significance for clinical decision-making. Currently, there is a lack of simple, intuitive, and externally validated individualized prognostic prediction tools in clinical practice.

**Objective:**

To develop and validate a nomogram model for predicting prognosis in patients with SA-AKI based on clinically accessible indicators.

**Methods:**

A retrospective case–control study design was employed. Clinical data were collected from 200 patients with SA-AKI hospitalized at The First Affiliated Hospital of Xinjiang Medical University and The Fifth Affiliated Hospital of Xinjiang Medical University from December 2023 to March 2025. Data from The First Affiliated Hospital of Xinjiang Medical University served as the training set (*n* = 113), while data from The Fifth Affiliated Hospital of Xinjiang Medical University served as the external validation set (*n* = 87). Key predictive variables were selected using least absolute shrinkage and selection operator (LASSO) regression and subsequently incorporated into multivariate logistic regression analysis to identify independent influencing factors, based on which a nomogram prediction model was constructed. Discrimination of the model was assessed using the receiver operating characteristic curve, calibration was evaluated using the Hosmer–Lemeshow test, and clinical utility was assessed using decision curve analysis.

**Results:**

LASSO regression identified four variables: age, Sequential Organ Failure Assessment (SOFA) score, creatinine, and serum potassium. Multivariate logistic regression analysis showed that all four variables were independent influencing factors for the prognosis of SA-AKI patients (all *p* < 0.01). Higher levels of age, SOFA score, creatinine, and serum potassium were significantly associated with an unfavorable prognosis: age (OR = 0.94, 95% CI: 0.91–0.97), SOFA score (OR = 0.75, 95% CI: 0.67–0.84), creatinine (OR = 0.60, 95% CI: 0.39–0.91), and serum potassium (OR = 0.51, 95% CI: 0.32–0.80). The nomogram model achieved an AUC of 0.92 (95% CI: 0.86–0.97) in the training set, with a sensitivity of 0.82 and a specificity of 0.90; in the external validation set, the AUC was 0.85 (95% CI: 0.76–0.94), with a sensitivity of 0.90 and a specificity of 0.66. The Hosmer–Lemeshow test indicated good calibration in both the training set (*p* = 0.41) and the validation set (*p* = 0.57), and decision curve analysis confirmed the clinical utility of the model.

**Conclusion:**

Age, SOFA score, creatinine, and serum potassium are independent predictors of prognosis in SA-AKI patients. The nomogram model constructed based on these indicators demonstrates good discrimination, calibration, and clinical utility, and may serve as a valuable tool for early identification of high-risk patients with poor prognosis and for the development of individualized treatment strategies.

## 1 Background

Sepsis is a life-threatening organ dysfunction caused by a dysregulated host response to infection, and is associated with extremely high morbidity and mortality (1). Globally, there are approximately 48.9 million cases of sepsis annually, with 30–50% progressing to septic shock (2, 3). Acute kidney injury (AKI) is one of the most common and serious complications of sepsis, occurring in approximately 60–70% of patients with septic shock (4). Sepsis-associated acute kidney injury (SA-AKI) is significantly associated with increased mortality, prolonged intensive care unit stay, increased requirement for renal replacement therapy, and an elevated risk of long-term chronic kidney disease. The in-hospital mortality rate of SA-AKI patients is as high as 40–60%, and approximately 30% of survivors will progress to chronic kidney disease (4, 5). Therefore, early identification of high-risk patients with SA-AKI and accurate prediction of their prognosis are of great significance for guiding clinical decision-making and improving patient outcomes.

Currently, the assessment of disease severity and prognosis prediction in patients with SA-AKI in clinical practice primarily relies on comprehensive scoring systems such as the Sequential Organ Failure Assessment (SOFA) score, along with certain laboratory indicators. However, these tools are mostly used to evaluate overall disease severity, and there remains a lack of simple, intuitive, and visualized prediction tools that can directly provide individual patients with specific prognostic probabilities. As an intuitive risk prediction model, the nomogram can transform multivariate regression results into a graphical scoring system, providing clinicians with individualized risk predictions, and has been widely applied in various diseases (6–8). Nevertheless, in the field of SA-AKI, studies on prediction models for clinical prognosis remain relatively limited. Existing models predominantly focus on the risk of AKI occurrence during early admission, whereas prediction models specifically targeting the prognosis of patients with confirmed SA-AKI are scarce. Moreover, most of these models are based on single-center studies lacking external validation, thereby limiting their clinical applicability. For instance, Jiang et al. developed a nomogram model for predicting the risk of septic shock-associated AKI, which was internally validated; however, the study focused on AKI occurrence rather than the prognosis of patients with confirmed SA-AKI, and it was not validated in an independent external center (9). Similarly, Wang and Zhang developed a prediction model for respiratory failure in patients with SA-AKI, which also focused on complication risk rather than overall patient prognosis and lacked independent external validation (10). Additionally, existing models predominantly employ traditional stepwise regression for variable selection, which carries a risk of overfitting and involves a relatively large number of variables, thus limiting their clinical operability.

To address this, the present study adopted a retrospective case–control design, collecting clinical and laboratory indicators of SA-AKI patients at the time of outcome occurrence. Least absolute shrinkage and selection operator (LASSO) was employed to identify key predictors, followed by the construction of a multivariate logistic regression model and the development of a nomogram. The aim was to establish a simple and effective prognostic prediction tool for SA-AKI patients, thereby providing a reference for clinicians to facilitate early identification of high-risk patients with poor prognosis and to formulate individualized treatment strategies.

## 2 Materials and methods

### 2.1 Study design and data sources

This study employed a retrospective case–control design. Clinical data were collected from patients with SA-AKI who were hospitalized in the intensive care unit (ICU) and respiratory intensive care unit (RICU) of The First Affiliated Hospital of Xinjiang Medical University and The Fifth Affiliated Hospital of Xinjiang Medical University from December 2023 to March 2025. Based on their in-hospital prognosis, patients were divided into a favorable outcome group (improved) and an unfavorable outcome group (death/deterioration). A total of 200 patients with SA-AKI were enrolled, including 87 cases (43.50%) in the favorable outcome group and 113 cases (56.50%) in the unfavorable outcome group. The data collection process adhered to the Transparent Reporting of a Multivariable Prediction Model for Individual Prognosis or Diagnosis (TRIPOD) statement to ensure the standardization and reproducibility of the study (11). This study was approved by the Ethics Committee of Xinjiang Medical University (approval no.: K202508-09). All patient information was de-identified prior to analysis, in compliance with medical ethics requirements. To ensure consistency across the two hospitals, an identical standardized case report form was used for data extraction at both sites, and all data collectors received the same training on the extraction protocol before the study commenced.

### 2.2 Study subjects

#### 2.2.1 Inclusion and exclusion criteria

Inclusion criteria: (1) age ≥18 years; (2) meeting the Sepsis-3.0 diagnostic criteria for sepsis (12): (1) presence of confirmed or suspected infection; (2) SOFA score ≥2 points; (3) meeting the AKI diagnostic criteria of the Kidney Disease: Improving Global Outcomes (KDIGO) guidelines (13): (1) an increase in serum creatinine by ≥26.5 μmol/L (0.3 mg/dL) within 48 h; (2) an increase in serum creatinine to ≥1.5 times the baseline value, which is known or presumed to have occurred within the previous 7 days; (2) urine output <0.5 mL/kg/h for >6 h.

Exclusion criteria: (1) patients with AKI caused by confirmed or suspected prerenal causes, postrenal causes, acute glomerulonephritis, acute interstitial nephritis, renal vasculitis, or other chronic kidney diseases; (2) patients with pre-existing chronic renal insufficiency (estimated glomerular filtration rate <30 mL/min/1.73 m^2^), end-stage renal disease, end-stage of various diseases, or irreversible terminal conditions; (3) patients with concomitant malignant tumors, immunodeficiency diseases, hematologic diseases, or infectious diseases; (4) patients who had used nephrotoxic drugs within 1 month prior to admission; (5) patients who had undergone kidney transplantation; (6) patients with a hospital stay <24 h; (7) pregnant women and women within 42 days postpartum; (8) patients with missing clinical data.

#### 2.2.2 Sample size estimation

This study was a retrospective case–control study, and the sample size estimation was performed with reference to the minimum sample size requirements for clinical prediction model development studies. According to the sample size calculation criteria for prediction models proposed by Riley et al., the number of outcome events should be at least 10 to 20 per candidate predictor variable to ensure the reliability of model development (14). LASSO regression was employed for predictor variable selection in this study, a method that can effectively control overfitting even with a relatively small sample size. Based on the expected inclusion of 10–15 candidate predictor variables and an anticipated events-per-variable ratio of 10 or greater, the minimum required number of outcome events was estimated to be 100–150. With 113 events observed in the unfavorable outcome group, the sample size of this study was considered adequate for prediction model development.

### 2.3 Research methods

#### 2.3.1 Data collection

Clinical data of patients were collected through the hospital electronic medical record system, covering demographic characteristics, underlying diseases, clinical scores, laboratory indicators, and prognostic outcomes. Demographic data included age and sex. Underlying diseases recorded included hypertension, diabetes mellitus, and coronary heart disease. Lifestyle factors collected included smoking history and alcohol consumption history. The clinical score used was the SOFA score based on the worst values within 24 h of admission. Laboratory indicators were the results of the first tests performed within 24 h of admission, including creatinine, serum potassium, bicarbonate, D-dimer, troponin, direct bilirubin, alanine aminotransferase (ALT), aspartate aminotransferase (AST), red blood cells, and platelets. All tests were completed by the hospital clinical laboratory using standardized methods. The components and scoring criteria of the SOFA score are shown in Table 1.

**Table 1:** Components and scoring criteria of the sequential organ failure assessment score.

Organ system	Variable	0 Point	1 Point	2 Points	3 Points	4 Points
Respiratory	PaO₂/FiO₂ (mmHg)	≥400	<400	<300	<200 (with respiratory support)	<100 (with respiratory support)
Coagulation	Platelets (×10^9^/L)	≥150	<150	<100	<50	<20
Liver	Bilirubin (μmol/L)	<20	20–32	33–101	102–204	>204
Cardiovascular	Mean arterial pressure (MAP) or vasopressor requirement	MAP ≥70 mmHg	MAP <70 mmHg	Dopamine ≤5 or dobutamine (any dose)	Dopamine >5 or epinephrine ≤0.1 or norepinephrine ≤0.1	Dopamine >15 or epinephrine >0.1 or norepinephrine >0.1
Central nervous system	Glasgow Coma Scale (GCS) score	15	13–14	10–12	6–9	<6
Renal	Creatinine (μmol/L) or urine output	<110	110–170	171–299	300–440 or urine output <500 mL/d	>440 or urine output <200 mL/d

PaO_2_/FiO_2_, ratio of arterial partial pressure of oxygen to fraction of inspired oxygen; MAP, mean arterial pressure; GCS, Glasgow Coma Scale. Vasopressor doses are expressed in μg/kg/min.

#### 2.3.2 Outcome assessment

The primary outcome variable of this study was the in-hospital prognosis. The criteria for outcome assessment were as follows:

Favorable outcome (favorable outcome group): patients who achieved improvement in clinical symptoms, recovery of renal function to baseline or stabilization, met discharge criteria, and did not experience in-hospital death.

Unfavorable outcome (unfavorable outcome group): patients who met any of the following criteria: (1) persistent deterioration of renal function, characterized by continuous increase in serum creatinine or persistent decrease in urine output; (2) initiation of renal replacement therapy (including continuous renal replacement therapy, intermittent hemodialysis, etc.) due to worsening renal function; (3) in-hospital death; (4) death confirmed by follow-up after automatic discharge. Follow-up was primarily conducted through electronic medical record system queries, supplemented by telephone follow-up, with a follow-up success rate of 92.3%.

To ensure objectivity and consistency of outcome assessment, two trained clinicians independently assessed the outcomes based on electronic medical records. If the assessment results were inconsistent, a third senior clinician served as an arbiter, and the final result was based on the arbitration opinion. The assessors were blinded to the predictor variables of this study prior to assessment to minimize assessment bias to the greatest extent possible.

#### 2.3.3 Bias control

Considering that the retrospective design might be subject to selection bias, information bias, and confounding bias, the following control measures were employed in this study:

1. Uniform inclusion and exclusion criteria were adopted to ensure the homogeneity of the study subjects.
2. All variables were clearly defined, and data extraction was independently performed by two researchers, followed by cross-verification.
3. The missing rate for key variables was <5%. Continuous variables were imputed using the median, and categorical variables were imputed using the mode. Variables with a missing rate >10% were excluded.
4. Potential confounders were adjusted for in the multivariate regression model.

### 2.4 Statistical analysis

Statistical analyses were performed using R software (version 4.2.0). Data that were not normally distributed were expressed as median (Q_1_, Q_3_), and comparisons between two groups were conducted using the Mann–Whitney *U* test. Categorical data were presented as frequencies, and comparisons between groups were performed using the Chi-square test. LASSO regression was employed to screen potential influencing factors, aiming to reduce the risk of overfitting and identify key variables. The penalty parameter *λ* in LASSO regression was selected using ten-fold cross-validation based on the “1-SE criterion” (i.e., selecting the largest λ value within one standard error of the minimum cross-validation error), to achieve a balance between model goodness-of-fit and parsimony. The variables selected by LASSO were incorporated into a multivariate logistic regression model to analyze the independent influencing factors for prognosis in SA-AKI patients, and odds ratios (ORs) with 95% confidence intervals (CIs) were calculated. Data from The First Affiliated Hospital of Xinjiang Medical University served as the training set, while data from The Fifth Affiliated Hospital of Xinjiang Medical University served as the validation set. The prediction model was constructed in the training set and externally validated in the validation set. Discriminative ability of the model was assessed using the receiver operating characteristic (ROC) curve, and the area under the curve (AUC) was calculated. Model calibration was evaluated using the Hosmer–Lemeshow goodness-of-fit test.

Decision curve analysis (DCA) was used to evaluate the clinical net benefit and utility of the nomogram model. The analysis was performed using the “rmda” package in R software. DCA calculates the net benefit at each threshold probability using the following formula: $\mathrm{Net}\;\text{Benefit}=\frac{\text{True Positives}}{n}–\frac{\text{False Positives}}{n}\times \frac{P_{t}}{1-P_{t}\;}$ , where P_t_ is the threshold probability (i.e., the minimum risk level at which a clinician would choose to intervene), and n is the total number of patients. The threshold probabilities analyzed ranged from 0 to 1, with increments of 0.01, covering clinical decision scenarios from “intervention at very low risk” to “intervention only at very high risk.” The net benefit curve of the nomogram was compared with two extreme strategies: “intervention for all” (assuming intervention for every patient) and “intervention for none” (assuming no intervention for any patient). The nomogram was considered clinically useful when its net benefit exceeded that of both extreme strategies within a specific range of threshold probabilities.

A two-tailed test was used, and a *p*-value < 0.05 was considered statistically significant.

## 3 Results

### 3.1 Comparison of factors between the favorable and unfavorable outcome groups of SA-AKI in the training set

A total of 113 patients with SA-AKI were included in the training set, comprising 41 cases (36.28%) in the favorable outcome group and 72 cases (63.72%) in the unfavorable outcome group. Univariate analysis revealed statistically significant differences between the two groups in age, SOFA score, creatinine, serum potassium, D-dimer, direct bilirubin, ALT, AST, and sex (all *p* < 0.05). Specifically, patients in the unfavorable outcome group had significantly higher levels of age, SOFA score, creatinine, serum potassium, D-dimer, and direct bilirubin compared with those in the favorable outcome group (*p* < 0.01); conversely, ALT and AST levels were lower in the unfavorable outcome group than in the favorable outcome group (*p* < 0.01). Regarding sex distribution, the proportion of males in the unfavorable outcome group (66.67%) was higher than that in the favorable outcome group (43.90%), with a statistically significant difference (*p* = 0.018). No statistically significant differences were observed between the two groups in bicarbonate, troponin, red blood cells, platelets, hypertension, diabetes mellitus, coronary heart disease, smoking history, or alcohol consumption history (all *p* > 0.05). Detailed results are shown in Table 2.

**Table 2:** Comparison of factors between the favorable and unfavorable outcome groups of SA-AKI.

Variables	Total (*n* = 113)	Unfavorable outcome group (*n* = 72)	Favorable outcome group (*n* = 41)	Statistic	*p*
Age (years)	77.00 (61.00, 83.00)	80.50 (74.00, 84.25)	62.00 (53.00, 73.00)	*Z* = −5.15	<0.001
SOFA score (points)	9.00 (5.00, 11.00)	10.00 (8.00, 12.00)	5.00 (3.00, 6.00)	*Z* = −5.69	<0.001
Creatinine (mg/dL)	1.50 (0.85, 1.87)	1.65 (0.92, 1.96)	1.05 (0.85, 1.44)	*Z* = −2.99	0.003
serum potassium (mmol/L)	4.19 (3.51, 5.31)	4.79 (4.00, 5.31)	3.64 (3.17, 4.02)	*Z* = −5.40	<0.001
Bicarbonate (mmol/L)	21.25 (18.06, 24.40)	21.10 (17.99, 24.30)	22.63 (18.47, 24.57)	*Z* = −0.42	0.676
D-dimer (mg/L)	2.77 (1.16, 5.49)	3.20 (2.02, 6.00)	1.97 (0.66, 4.64)	*Z* = −2.57	0.010
Troponin (μg/L)	0.05 (0.02, 0.14)	0.06 (0.03, 0.14)	0.03 (0.01, 0.17)	*Z* = −1.67	0.096
Direct bilirubin (μmol/L)	7.10 (0.30, 14.35)	8.90 (4.60, 18.00)	0.30 (0.30, 9.40)	*Z* = −4.41	<0.001
ALT(U/L)	29.70 (16.93, 55.00)	23.07 (12.39, 42.35)	42.09 (27.00, 77.79)	*Z* = −3.77	<0.001
AST (U/L)	42.35 (27.70, 81.30)	34.85 (21.57, 63.88)	49.17 (36.12, 116.36)	*Z* = −3.16	0.002
Red blood cells (×10^12^/L)	3.82 (3.19, 4.57)	3.90 (3.10, 4.63)	3.67 (3.30, 4.44)	*Z* = −0.01	0.993
Platelets (×10^9^/L)	159.00 (110.00, 232.00)	166.00 (122.50, 257.50)	142.00 (95.00, 199.00)	*Z* = −1.80	0.072
Sex				*χ*^2^ = 5.57	0.018
Male	66 (58.41)	48 (66.67)	18 (43.90)		
Female	47 (41.59)	24 (33.33)	23 (56.10)		
Hypertension				*χ*^2^ = 0.02	0.901
No	57 (50.44)	36 (50.00)	21 (51.22)		
Yes	56 (49.56)	36 (50.00)	20 (48.78)		
Diabetes mellitus				*χ*^2^ = 2.46	0.117
No	75 (66.37)	44 (61.11)	31 (75.61)		
Yes	38 (33.63)	28 (38.89)	10 (24.39)		
Coronary heart disease				*χ*^2^ = 2.46	0.117
No	81 (71.68)	48 (66.67)	33 (80.49)		
Yes	32 (28.32)	24 (33.33)	8 (19.51)		
Smoking history				*χ*^2^ = 0.01	0.927
No	96 (84.96)	61 (84.72)	35 (85.37)		
Yes	17 (15.04)	11 (15.28)	6 (14.63)		
Alcohol consumption history				*χ*^2^ = 0.23	0.631
No	100 (88.50)	65 (90.28)	35 (85.37)		
Yes	13 (11.50)	7 (9.72)	6 (14.63)		

### 3.2 Screening of potential influencing factors for prognosis in SA-AKI patients

**Figure 1:**
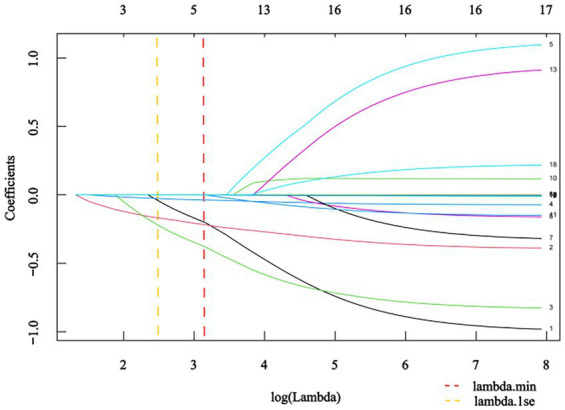
LASSO regression coefficient paths. The plot displays the LASSO coefficient paths of 18 candidate variables against log(λ). Each curve represents the trajectory of an individual variable’s coefficient as the penalty parameter λ varies. The left dashed line corresponds to λ 1-SE (at which four variables had non-zero coefficients), and the right dashed line corresponds to λ min (at which six variables had non-zero coefficients). Variables with non-zero coefficients at the optimal λ were retained for further analysis.

**Figure 2:**
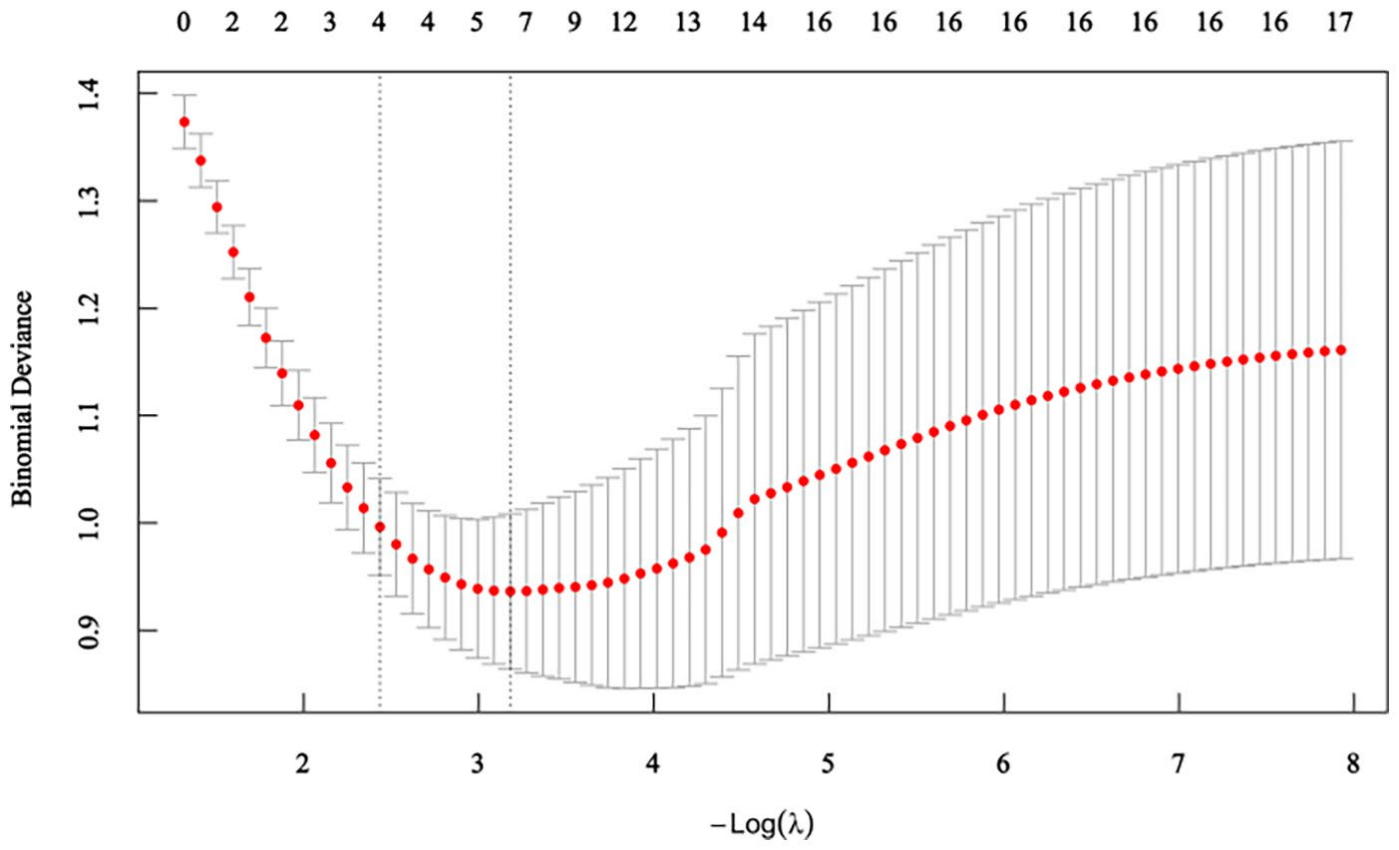
Ten-fold cross-validation curve of LASSO regression. The left vertical dashed line indicates the λ value that yields the minimum cross-validation error (λ min), under which six variables were selected; the right vertical dashed line indicates the largest λ within one standard error of the minimum (λ 1-SE), under which four variables were selected. The optimal λ was chosen according to the 1-SE criterion, with four variables (creatinine, SOFA score, potassium, and age) ultimately retained to achieve a balance between model goodness-of-fit and parsimony. The dotted lines represent the cross-validation error ± one standard error.

All factors presented in Table 2 were included as independent variables, and the patient prognosis outcome (favorable outcome = 1, unfavorable outcome = 0) was used as the dependent variable. LASSO regression was employed for variable selection. The optimal penalty parameter λ was determined using ten-fold cross-validation. At λ min (the λ value yielding the minimum cross-validation error), six variables were retained, whereas at λ 1-SE (the largest λ within one standard error of the minimum), four variables were selected. The optimal λ was chosen according to the 1-SE criterion to achieve a balance between model goodness-of-fit and parsimony. Ultimately, four potential influencing factors significantly associated with unfavorable prognosis were identified: creatinine, SOFA score, serum potassium, and age. The variable selection path plot and the cross-validation error curve for LASSO regression are shown in Figures 1, 2, respectively.

### 3.3 Multivariate logistic regression analysis of prognosis in SA-AKI patients

The four variables selected by LASSO regression (creatinine, SOFA score, serum potassium, and age) were included as independent variables, and patient prognosis outcome (favorable outcome = 1, unfavorable outcome = 0) was used as the dependent variable in a multivariate logistic regression analysis. The results showed that all four variables were independent influencing factors for the prognosis of SA-AKI patients (all *p* < 0.01). Specifically, creatinine (OR = 0.60, 95% CI: 0.39–0.91, *p* = 0.018), SOFA score (OR = 0.75, 95% CI: 0.67–0.84, *p* < 0.001), serum potassium (OR = 0.51, 95% CI: 0.32–0.80, *p* = 0.003), and age (OR = 0.94, 95% CI: 0.91–0.97, *p* < 0.001) were all negatively associated with prognosis, indicating that higher levels of these indicators were associated with a lower likelihood of a favorable outcome in patients. Detailed results are shown in Table 3.

**Table 3:** Multivariate logistic regression analysis of factors associated with prognosis in SA-AKI patients.

Variables	β	S.E.	*Z*	*p*	OR (95% CI)
Creatinine (mg/dL)	−0.52	0.22	−2.37	0.018	0.60 (0.39 ~ 0.91)
SOFA score (points)	−0.28	0.06	−4.81	< 0.001	0.75 (0.67 ~ 0.84)
Serum potassium (mmol/L)	−0.67	0.23	−2.93	0.003	0.51 (0.32 ~ 0.80)
Age (years)	−0.06	0.01	−4.31	< 0.001	0.94 (0.91 ~ 0.97)

### 3.4 Construction of a prognostic prediction model for SA-AKI patients

**Figure 3:**
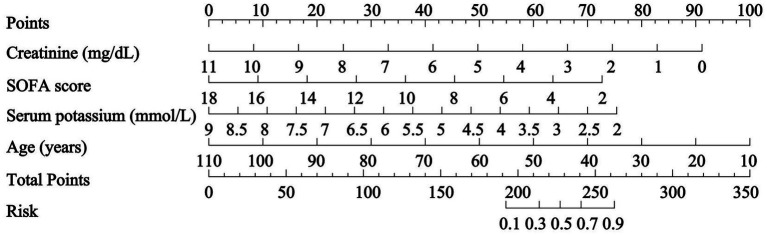
Nomogram of the prognostic prediction model for SA-AKI patients. To use the nomogram, locate each variable on its respective axis, draw a vertical line upward to the “Points” scale to obtain the assigned score, and sum the scores for all four variables. The total points correspond to the predicted probability of a favorable outcome on the lowest scale. Higher total points indicate a lower likelihood of a favorable prognosis. The four predictor variables included in the model are age (years), SOFA score, creatinine (mg/dL), and serum potassium (mmol/L). For example, consider a hypothetical patient aged 50 years (60 points), with a SOFA score of 8 (45 points), creatinine of 2.0 mg/dL (75 points), and serum potassium of 3.0 mmol/L (65 points). The total score would be 240 points, corresponding to a predicted probability of a favorable outcome of approximately 0.70.

A nomogram model was constructed based on the four aforementioned predictive variables. In this model, a vertical line is drawn from the value of each variable to the scale line at the top to obtain the corresponding points. The points for each variable are summed to obtain the total score, which corresponds to the risk scale at the bottom. A higher total score indicates a stronger association with the risk of death or deterioration in SA-AKI patients (Figure 3).

### 3.5 Model performance evaluation

**Figure 4:**
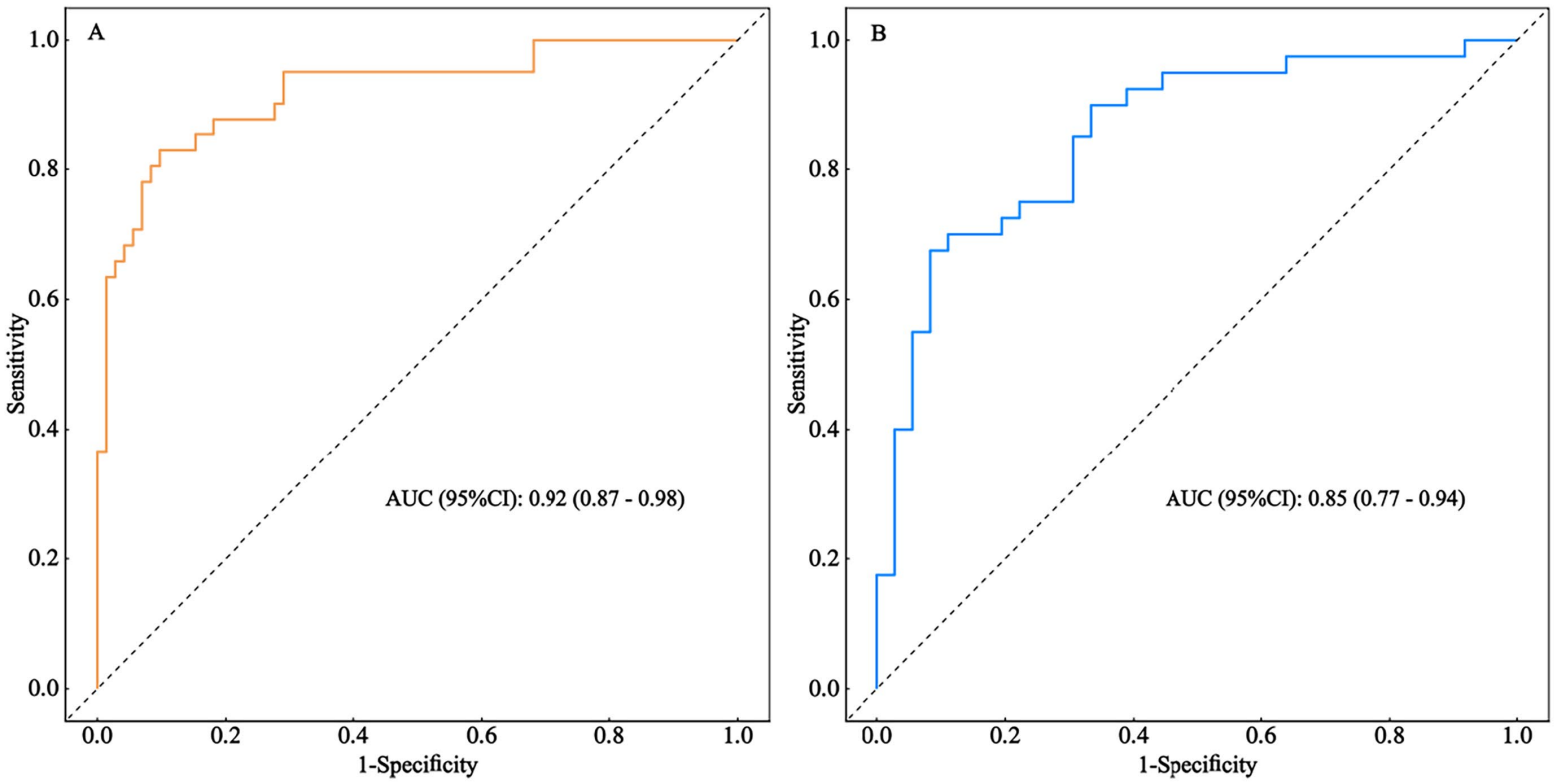
ROC curves of the model. **(A)** ROC curve of the training set; **(B)** ROC curve of the validation set. The diagonal reference line represents an AUC of 0.5, indicating no discriminative ability.

**Figure 5:**
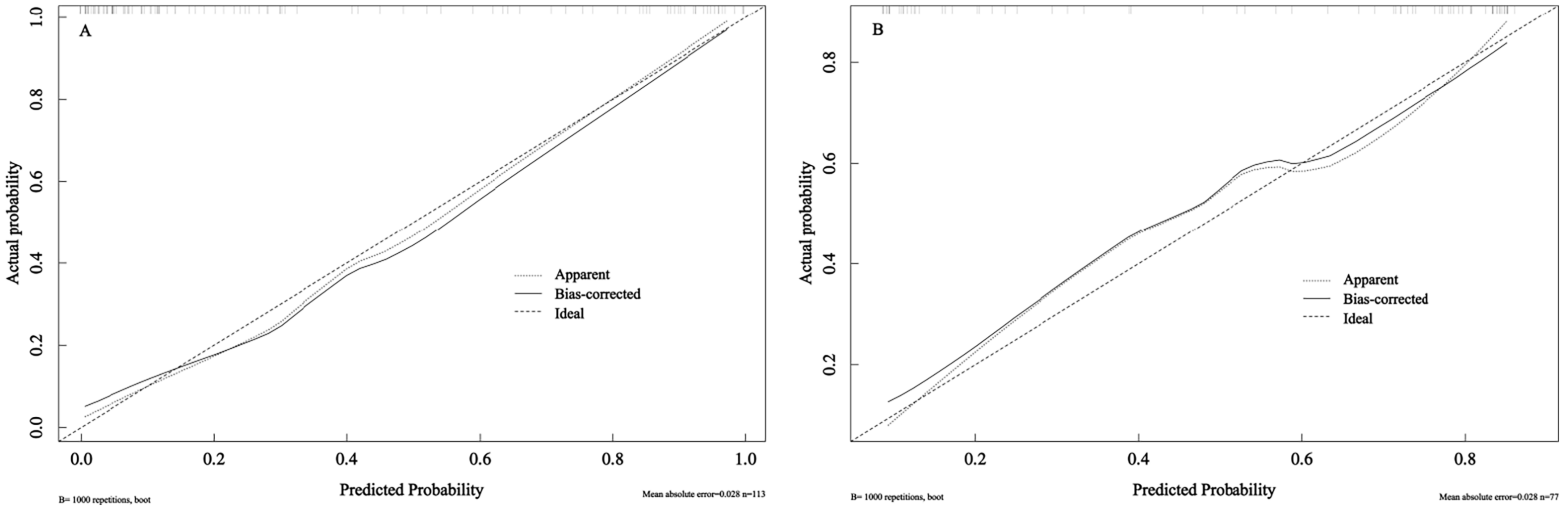
Calibration curves of the model. **(A)** Calibration curve of the training set. **(B)** Calibration curve of the validation set. The x-axis represents the predicted probability of a favorable outcome, and the y-axis represents the actual observed probability of a favorable outcome. The diagonal dashed line represents perfect calibration (ideal prediction). The solid line represents the calibration performance of the model.

**Figure 6:**
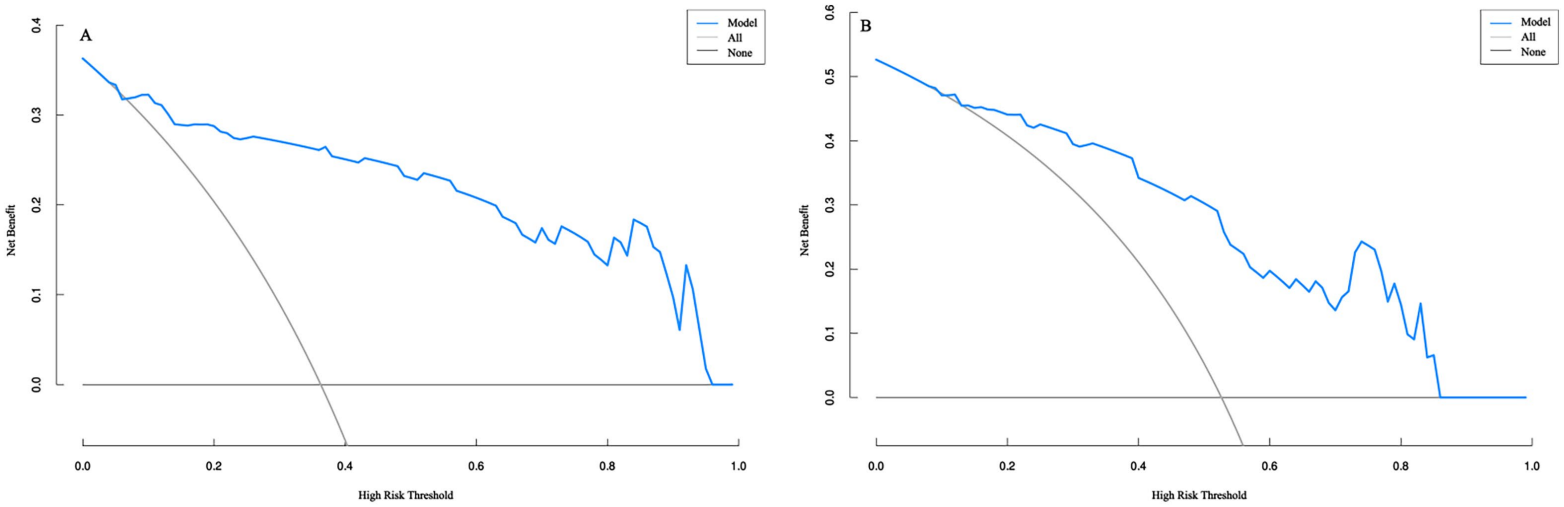
DCA curves of the model. **(A)** DCA curve of the training set. **(B)** DCA curve of the validation set. The x-axis represents the threshold probability, and the y-axis represents the net benefit. The horizontal solid line at y = 0 represents the “intervention for none” strategy, and the grey solid line represents the “intervention for all” strategy. The blue solid line represents the net benefit of the nomogram-guided intervention strategy.

The nomogram model achieved an AUC of 0.92 (95% CI: 0.86–0.97) in the training set, with a sensitivity of 0.82 and a specificity of 0.90; in the external validation set, the model achieved an AUC of 0.85 (95% CI: 0.76–0.94), with a sensitivity of 0.90 and a specificity of 0.66, indicating good discriminative ability. The calibration curves in both the training set and the external validation set showed good agreement with the ideal curve. The Hosmer–Lemeshow goodness-of-fit test yielded no statistical significance for either the training set (*χ*^2^ = 8.215, df = 8, *p* = 0.413) or the external validation set (*χ*^2^ = 6.680, df = 8, *p* = 0.572), indicating good calibration of the model. Regarding clinical utility, decision curve analysis showed that the net benefit of the model was higher than the strategies of “no intervention” or “intervention for all patients” within a certain range of threshold probabilities, demonstrating good clinical applicability of the model (Figures 4–6; Table 4).

**Table 4:** Performance evaluation of the prognostic prediction model for SA-AKI patients.

Data	AUC (95% CI)	Accuracy (95% CI)	Sensitivity (95% CI)	Specificity (95% CI)	PPV (95% CI)	NPV (95% CI)	Cut off
Train	0.921 (0.867–0.976)	0.876 (0.801–0.931)	0.829 (0.714–0.944)	0.903 (0.834–0.971)	0.829 (0.714–0.944)	0.903 (0.834–0.971)	0.411
Test	0.855 (0.769–0.941)	0.789 (0.681–0.875)	0.900 (0.807–0.993)	0.667 (0.513–0.821)	0.750 (0.628–0.872)	0.857 (0.728–0.987)	0.411

## 4 Discussion

This study employed a retrospective case–control design and included 200 patients with SA-AKI. Four key predictive variables—age, SOFA score, creatinine, and serum potassium—were identified via LASSO regression, and a nomogram model for predicting patient prognosis was constructed. Multivariate logistic regression analysis showed that all four variables were independent influencing factors for the prognosis of SA-AKI patients (all *p* < 0.01), and all were significantly associated with prognosis outcome. Specifically, older age, higher SOFA score, higher creatinine level, and higher serum potassium level were associated with a lower likelihood of a favorable outcome. The model demonstrated good discriminative ability in both the training set and the external validation set (with AUC values of 0.92 and 0.85, respectively) and good calibration (Hosmer–Lemeshow test *p* > 0.05). Decision curve analysis further confirmed its favorable clinical utility.

In this study, LASSO regression was employed for variable selection. This method introduces an L1 penalty term into the regression model, which can compress the coefficients of irrelevant variables to zero, thereby achieving simultaneous variable selection and parameter estimation. Compared with traditional stepwise regression, LASSO regression effectively controls the risk of overfitting, particularly in situations with a limited sample size and a large number of candidate variables, thereby improving model stability and generalization ability (15, 16). It is noteworthy that although direct bilirubin showed a substantial difference between the favorable and unfavorable outcome groups in the univariate analysis (0.30 μmol/L vs. 8.90 μmol/L, *p* < 0.001), it was not retained in the final LASSO model. This can be explained by the fact that the SOFA score incorporates bilirubin as part of its liver component assessment, resulting in collinearity between direct bilirubin and the SOFA score. LASSO regression employs an L1 penalty that automatically shrinks the coefficients of strongly correlated variables toward zero, retaining only the variable with stronger independent predictive power (in this case, the SOFA score). This illustrates one of the advantages of LASSO regression in handling multicollinearity among candidate predictors and selecting a more parsimonious set of variables.

This study found that age was an independent predictor of prognosis in SA-AKI patients (OR = 0.94, 95% CI: 0.91–0.97, *p* < 0.001), with older age associated with a lower likelihood of a favorable outcome. This finding is highly consistent with those of previous studies. A large retrospective study conducted by Zeng et al. based on the MIMIC-IV database (*n* = 3,338) showed that age ≥ 76 years was an independent risk factor for 28-day mortality in elderly patients with SA-AKI (HR = 1.39, 95% CI: 1.21–1.59, *p* < 0.001) (17). A meta-analysis comprising 18 studies also demonstrated that age was an independent risk factor for mortality in SA-AKI patients (OR = 1.03, 95% CI: 1.01–1.05) (18). From a pathophysiological perspective, older patients exhibit a physiological decline in glomerular filtration rate with age, significantly reduced renal reserve capacity, along with impaired vascular endothelial function and diminished regulation of inflammatory responses, thereby resulting in markedly decreased tolerance to septic insults (19, 20). In addition, older patients often present with multiple underlying comorbidities, further increasing the risk of multiple organ dysfunction (21). Therefore, age, as an unmodifiable individual characteristic, should serve as an important basis for risk stratification in patients with SA-AKI.

The SOFA score also demonstrated a significant negative association with the prognosis of SA-AKI patients in this study (OR = 0.75, 95% CI: 0.67–0.84, *p* < 0.001), indicating that higher disease severity was associated with a lower probability of improvement. As an internationally recognized tool for assessing the degree of organ dysfunction in patients with sepsis, the association between the SOFA score and prognosis has been validated in numerous studies (22, 23). A review by Kounatidis et al. clearly indicated that the SOFA score is an important tool for assessing disease severity and predicting prognosis in SA-AKI patients (24). A meta-analysis by Lu et al. demonstrated that the SOFA score has good predictive performance for mortality in patients with sepsis, with a pooled AUC of 0.81 (95% CI: 0.78–0.84) (25). The study by Poston and Koyner also confirmed that the SOFA score is one of the important independent factors for predicting in-hospital mortality in SA-AKI patients (26). The SOFA score encompasses the functional status of six organ systems—respiratory, coagulation, liver, cardiovascular, central nervous system, and renal—and can comprehensively reflect the impact of sepsis on multiple organs (27). Patients with SA-AKI often present with concurrent dysfunction of other organs. A higher SOFA score indicates more severe organ failure, poorer compensatory capacity, and greater difficulty in clinical management (28). Therefore, the SOFA score serves not only as a core indicator for assessing disease severity but also as an important tool for predicting patient prognosis.

In this study, creatinine level was significantly negatively correlated with the prognosis of SA-AKI patients (OR = 0.60, 95% CI: 0.39–0.91, *p* = 0.018), indicating that higher creatinine levels were associated with a lower probability of a favorable outcome. A study by Zhang et al. confirmed that serum creatinine levels in SA-AKI patients were significantly higher than those in sepsis patients without AKI, and that creatinine levels were significantly higher in non-survivors than in survivors (29). Multiple studies have also demonstrated that elevated creatinine at admission is an independent risk factor for mortality in SA-AKI patients, with those having creatinine ≥1.5 mg/dL exhibiting significantly higher 28-day mortality rates (30, 31). From a pathophysiological perspective, creatinine level directly reflects glomerular filtration function; a significant increase in creatinine suggests severe injury to renal tubular epithelial cells and depletion of renal functional reserve (32). During the pathological process of SA-AKI, renal tubular epithelial cells are subjected to multiple insults, including ischemia–reperfusion injury, inflammatory cytokine attack, and mitochondrial dysfunction. In severe cases, apoptosis or necrosis may occur, leading to difficulty in renal function recovery (13, 33).

Serum potassium level was also identified as an independent predictor of prognosis in SA-AKI patients in this study (OR = 0.51, 95% CI: 0.32–0.80, *p* = 0.003), with hyperkalemia significantly increasing the risk of unfavorable outcomes. Electrolyte disturbances are common complications in SA-AKI patients, among which hyperkalemia is the most critical. A retrospective cohort study based on the MIMIC-IV database, including 8,242 patients with AKI complicated by sepsis, demonstrated that for each 1 mmol/L increase in serum potassium level at admission, the 30-day mortality risk increased by 13% (HR = 1.13, 95% CI: 1.06–1.20); patients in the hyperkalemia group (≥4.5 mmol/L) had a 22% increased risk of mortality compared with those in the normokalemia group (HR = 1.22, 95% CI: 1.08–1.38) (34). The pathophysiological mechanisms of hyperkalemia mainly involve two aspects: first, impaired renal serum potassium excretion, as SA-AKI patients have decreased glomerular filtration rate and reduced urine output, leading to impaired serum potassium ion excretion; second, redistribution of serum potassium ions between intracellular and extracellular compartments, as acidosis induced by sepsis can promote the shift of serum potassium ions from the intracellular to the extracellular space, further exacerbating hyperkalemia (34). Hyperkalemia can cause electrophysiological abnormalities in myocardial cells, triggering malignant arrhythmias and even cardiac arrest, and is one of the important causes of death in SA-AKI patients (35, 36). Therefore, in the management of SA-AKI patients, serum potassium levels should be closely monitored, and hyperkalemia should be promptly corrected.

The nomogram model constructed in this study incorporates only four clinically accessible indicators (age, SOFA score, creatinine, and serum potassium), does not rely on expensive special tests, and thus demonstrates good clinical operability. The model exhibited good discriminative ability and calibration in the external validation set, suggesting favorable generalizability. The nomogram is presented in a visual format, allowing clinicians to rapidly calculate risk scores based on individual patient indicators, thereby enabling intuitive assessment of prognosis-related factors for individual patients. To further enhance clinical utility, a simplified scoring scale or electronic calculation tool could be developed based on this nomogram in the future, facilitating rapid bedside application by frontline clinicians. Furthermore, this model may serve as an adjunctive tool for early identification of high-risk patients and clinical decision-making, providing a reference for the formulation of individualized treatment strategies.

The present study has the following limitations. First, this study employed a retrospective case–control design, and exposure indicators were collected at the time of outcome occurrence rather than at a uniform baseline time point, introducing inherent temporal bias and making it impossible to completely exclude the influence of disease course stage on the indicators. Second, the data were derived from two hospitals, with a relatively limited sample size. Although external validation was performed, the sample size may still affect model stability, and future multicenter prospective studies are needed to further validate the generalizability of the model. Third, novel biomarkers (such as neutrophil gelatinase-associated lipocalin [NGAL] and kidney injury molecule-1 [KIM-1]) were not included for comparison. These biomarkers have high sensitivity in the early detection of kidney injury, and future studies could further explore their incremental value in enhancing the evaluative performance of this model. Fourth, due to data limitations, the performance of the model across different subgroups (such as different KDIGO stages or different sources of sepsis) could not be analyzed, and future research should further evaluate the applicability of the model in subgroups. Fifth, the analysis of long-term patient prognosis was not performed, and future prospective cohort studies are needed to more comprehensively validate this model.

## 5 Conclusion

In conclusion, age, SOFA score, creatinine, and serum potassium are independent predictors of prognosis in patients with SA-AKI. The nomogram model constructed based on these indicators demonstrates good discrimination, calibration, and clinical utility, and may serve as a valuable tool for early identification of high-risk patients with poor prognosis and for the formulation of individualized treatment strategies.

## Data Availability

The raw data supporting the conclusions of this article will be made available by the authors, without undue reservation.

## References

[1] Martin-Loeches I Singer M Leone M. Sepsis: key insights, future directions, and immediate goals. A review and expert opinion. Intensive Care Med. (2024) 50:2043–9. doi: 10.1007/s00134-024-07694-z, 39531053

[2] La Via L Maniaci A Lentini M Cuttone G Ronsivalle S Tutino S . The burden of sepsis and septic shock in the intensive care unit. J Clin Med. (2025) 14:6691. doi: 10.3390/jcm14196691, 41095771 PMC12525046

[3] Rudd KE Johnson SC Agesa KM Shackelford KA Tsoi D Kievlan DR . Global, regional, and national sepsis incidence and mortality, 1990–2017: analysis for the global burden of disease study. Lancet. (2020) 395:200–11. doi: 10.1016/S0140-6736(19)32989-7, 31954465 PMC6970225

[4] Takeuchi T Flannery AH Liu LJ Ghazi L Cama-Olivares A Fushimi K . Epidemiology of sepsis-associated acute kidney injury in the ICU with contemporary consensus definitions. Crit Care. (2025) 29:128. doi: 10.1186/s13054-025-05351-5, 40114218 PMC11924826

[5] Chiang H-Y Liang C-C Hsiao Y-L Le U-M Chang Y-C Chen P-S . Sepsis-associated acute kidney disease incidence, trajectory, and outcomes. Kidney Med. (2025) 7:100959. doi: 10.1016/j.xkme.2024.100959, 39990101 PMC11847305

[6] Meehan AJ Lewis SJ Fazel S Fusar-Poli P Steyerberg EW Stahl D . Clinical prediction models in psychiatry: a systematic review of two decades of progress and challenges. Mol Psychiatry. (2022) 27:2700–8. doi: 10.1038/s41380-022-01528-4, 35365801 PMC9156409

[7] Ogunpola A Saeed F Basurra S Albarrak AM Qasem SN. Machine learning-based predictive models for detection of cardiovascular diseases. Diagnostics. (2024) 14:144. doi: 10.3390/diagnostics14020144, 38248021 PMC10813849

[8] Wang L Liu Q Wu C Hou M Li Z. Establishment and validation of the prediction model based on lymphocyte subsets for acute kidney injury in sepsis patients. Front Immunol. (2025) 16:1674673. doi: 10.3389/fimmu.2025.1674673, 41080572 PMC12507742

[9] Jiang Z Hong S Chen Y Du C Hong Z Xie R . Development and validation of a prediction model for septic shock–associated acute kidney injury: a multicenter study using nomogram modeling. Shock. (2025) 64:311–21. doi: 10.1097/SHK.0000000000002631, 40550698

[10] Wang B Zhang F. Development and validation of a prediction model for respiratory failure in patients with Sepsis-associated acute kidney injury (SA-AKI) within 48 hours of admission. Emerg Med Int. (2025) 2025:5517872. doi: 10.1155/emmi/5517872, 41140320 PMC12552075

[11] Collins GS Reitsma JB Altman DG Moons KG. Transparent reporting of a multivariable prediction model for individual prognosis or diagnosis (TRIPOD): the TRIPOD statement. J Br Surg. (2015) 102:148–58. doi: 10.1002/bjs.9736, 25627261

[12] Singer M Deutschman CS Seymour CW Shankar-Hari M Annane D Bauer M . The third international consensus definitions for sepsis and septic shock (Sepsis-3). JAMA. (2016) 315:801–10. doi: 10.1001/jama.2016.0287, 26903338 PMC4968574

[13] Kellum JA Lameire N Aspelin P Barsoum RS Burdmann EA Goldstein SL . Kidney disease: improving global outcomes (KDIGO) acute kidney injury work group. KDIGO clinical practice guideline for acute kidney injury. Kidney Int Suppl. (2012) 2:1–138.

[14] Riley RD Ensor J Snell KI Harrell FE Martin GP Reitsma JB . Calculating the sample size required for developing a clinical prediction model. BMJ. (2020) 368:m441. doi: 10.1136/bmj.m441 32188600

[15] Liu L Jung S-H. Repeated sieving for prediction model building with high-dimensional data. J Pers Med. (2024) 14:769. doi: 10.3390/jpm14070769, 39064023 PMC11277592

[16] Tibshirani R. Regression shrinkage and selection via the lasso. J R Stat Soc Series B Stat Methodol. (1996) 58:267–88. doi: 10.1111/j.2517-6161.1996.tb02080.x

[17] Zeng J Zhang Y Zhang M Feng K Guo W Ge Y . Analysis of prognostic risk factors in critically ill elderly patients with sepsis-associated acute kidney injury. BMC Nephrol. (2025) 26:656. doi: 10.1186/s12882-025-04630-1, 41266989 PMC12632123

[18] Liu J Xie H Ye Z Li F Wang L. Rates, predictors, and mortality of sepsis-associated acute kidney injury: a systematic review and meta-analysis. BMC Nephrol. (2020) 21:318. doi: 10.1186/s12882-020-01974-8, 32736541 PMC7393862

[19] Fukuda M Fukami K Nabeta M Hirayu N Takasu O. Association of baseline renal function with mortality in patients with sepsis requiring continuous renal replacement therapy for acute kidney injury: a single-center retrospective study. Blood Purif. (2023) 52:148–56. doi: 10.1159/000525932, 36476403

[20] Michels EH Butler JM Reijnders TD Cremer OL Scicluna BP Uhel F . Association between age and the host response in critically ill patients with sepsis. Crit Care. (2022) 26:385. doi: 10.1186/s13054-022-04266-9, 36514130 PMC9747080

[21] Malla SS Kola VR Agiwal V Pant HB. Prevalence & risk factors of multi-morbidity in critically ill patients with sepsis-associated acute kidney injury (SA-AKI). Indian J Med Res. (2025) 161:665–71. doi: 10.25259/IJMR_2037_2024, 40878332 PMC12550376

[22] Ware LB Files DC Fowler A Aboodi MS Aggarwal NR Brower RG . Acetaminophen for prevention and treatment of organ dysfunction in critically ill patients with sepsis: the ASTER randomized clinical trial. JAMA. (2024) 332:390–400. doi: 10.1001/jama.2024.8772, 38762798 PMC11304120

[23] Yue S Li S Huang X Liu J Hou X Zhao Y . Machine learning for the prediction of acute kidney injury in patients with sepsis. J Transl Med. (2022) 20:215. doi: 10.1186/s12967-022-03364-0, 35562803 PMC9101823

[24] Kounatidis D Vallianou NG Psallida S Panagopoulos F Margellou E Tsilingiris D . Sepsis-associated acute kidney injury: where are we now? Medicina. (2024) 60:434. doi: 10.3390/medicina60030434, 38541160 PMC10971830

[25] Lu J Dong Z Ye L Gao Y Zheng Z. Predictive value of SOFA, PCT, lactate, qSOFA and their combinations for mortality in patients with sepsis: a systematic review and meta-analysis. PLoS One. (2025) 20:e0332525. doi: 10.1371/journal.pone.0332525, 40961067 PMC12443322

[26] Poston JT Koyner JL. Sepsis associated acute kidney injury. BMJ. (2019) 364:k4891. doi: 10.1136/bmj.k4891, 30626586 PMC6890472

[27] Vincent J-L Moreno R Takala J Willatts S De Mendonça A Bruining H . The SOFA (Sepsis-related organ failure assessment) score to describe organ dysfunction/failure: on behalf of the working group on Sepsis-related problems of the European Society of Intensive Care Medicine (see contributors to the project in the appendix). Intensive Care Med. (1996) 22:707–10. doi: 10.1007/BF01709751, 8844239

[28] Shin TG. Assessment of organ failure in sepsis patients in the emergency department: clinical evaluation, sequential organ failure assessment (SOFA) score, and future perspectives. Clin Exp Emerg Med. (2024) 11:327–30. doi: 10.15441/ceem.24.330, 39743307 PMC11700687

[29] Zhang Z Zhang Z Liu J Qiao L Fan X. Diagnostic value and prognostic significance of microRNA-210, serum creatinine, neutrophil gelatinase-associated lipocalin, blood urea nitrogen, cystatin C, and sequential organ failure assessment scores in patients with sepsis-associated acute kidney injury. Front Med. (2025) 12:1671599. doi: 10.3389/fmed.2025.1671599, 41567682 PMC12816257

[30] Lou J Xiang Z Zhu X Song J Cui S Li J . The non-linear association between creatinine-to-albumin ratio and medium-term mortality in patients with sepsis accompanied by acute kidney injury in the intensive care unit: a retrospective study based on the MIMIC database and external validation. Front Cell Infect Microbiol. (2025) 15:1602921. doi: 10.3389/fcimb.2025.1602921, 41425961 PMC12715007

[31] Ralib AM Ramly NF Nanyan S Nor MBM. The utility of the creatinine excretion to production ratio and the plasma creatinine and cystatin C based kinetic estimates of glomerular filtration rates in critically ill patients with Sepsis. Indian J Nephrol. (2022) 32:600–5. doi: 10.4103/ijn.ijn_519_21, 36704601 PMC9872923

[32] Deng R Yang H Zhong W Zhou J Huang G Zeng K. CITED2 mediates metabolic reprogramming in renal tubular epithelial cells via the AKT signaling pathway to induce Sepsis-associated acute kidney injury. J Inflamm Res. (2024) 17:9485–505. doi: 10.2147/JIR.S486596, 39600684 PMC11590677

[33] Pabla N Bajwa A. Role of mitochondrial therapy for ischemic-reperfusion injury and acute kidney injury. Nephron. (2022) 146:253–8. doi: 10.1159/000520698, 34883481 PMC9090938

[34] Guo Y Qiu Y Xue T Yan P Zhao W Wang M . Association between admission baseline blood potassium levels and all-cause mortality in patients with acute kidney injury combined with sepsis: a retrospective cohort study. PLoS One. (2024) 19:e0309764. doi: 10.1371/journal.pone.0309764, 39565797 PMC11578480

[35] El-Sherif N Turitto G. Electrolyte disorders and arrhythmogenesis. Cardiol J. (2011) 18:233–45. 21660912

[36] Zaza A. Serum potassium and arrhythmias. Europace. (2009) 11:421–2. doi: 10.1093/europace/eup005, 19182234

